# Creation of an Accurate Algorithm to Detect Snellen Best Documented Visual Acuity from Ophthalmology Electronic Health Record Notes

**DOI:** 10.2196/medinform.4732

**Published:** 2016-05-04

**Authors:** Michael Mbagwu, Dustin D French, Manjot Gill, Christopher Mitchell, Kathryn Jackson, Abel Kho, Paul J Bryar

**Affiliations:** ^1^ Department of Ophthalmology Northwestern University Feinberg School of Medicine Chicago, IL United States; ^2^ Institute for Public Health and Medicine Northwestern University Feinberg School of Medicine Chicago, IL United States; ^3^ Clinical and Translational Sciences Institute Northwestern University Chicago, IL United States

**Keywords:** visual acuity, best documented visual acuity, best corrected visual acuity, electronic health record, electronic medical record, phenotyping, data mining, ophthalmology

## Abstract

**Background:**

Visual acuity is the primary measure used in ophthalmology to determine how well a patient can see. Visual acuity for a single eye may be recorded in multiple ways for a single patient visit (eg, Snellen vs. Jäger units vs. font print size), and be recorded for either distance or near vision. Capturing the best documented visual acuity (BDVA) of each eye in an individual patient visit is an important step for making electronic ophthalmology clinical notes useful in research.

**Objective:**

Currently, there is limited methodology for capturing BDVA in an efficient and accurate manner from electronic health record (EHR) notes. We developed an algorithm to detect BDVA for right and left eyes from defined fields within electronic ophthalmology clinical notes.

**Methods:**

We designed an algorithm to detect the BDVA from defined fields within 295,218 ophthalmology clinical notes with visual acuity data present. About 5668 unique responses were identified and an algorithm was developed to map all of the unique responses to a structured list of Snellen visual acuities.

**Results:**

Visual acuity was captured from a total of 295,218 ophthalmology clinical notes during the study dates. The algorithm identified all visual acuities in the defined visual acuity section for each eye and returned a single BDVA for each eye. A clinician chart review of 100 random patient notes showed a 99% accuracy detecting BDVA from these records and 1% observed error.

**Conclusions:**

Our algorithm successfully captures best documented Snellen distance visual acuity from ophthalmology clinical notes and transforms a variety of inputs into a structured Snellen equivalent list. Our work, to the best of our knowledge, represents the first attempt at capturing visual acuity accurately from large numbers of electronic ophthalmology notes. Use of this algorithm can benefit research groups interested in assessing visual acuity for patient centered outcome. All codes used for this study are currently available, and will be made available online at https://phekb.org.

## Introduction

Visual acuity is one of the most important records of data in an ophthalmic examination. To an eye care provider, it is the equivalent of a vital sign, such as heart rate or blood pressure. In most electronic health records (EHRs), it is recorded as a free text in a defined field and not as pure structured data. Additionally, in a single clinical visit, visual acuity for a given eye may have several different values recorded within the EHR note. For example, a new patient seen by an ophthalmologist without correction (glasses) may see 20/100, with an old correction may see 20/30, but the “best corrected vision” with new glasses will see 20/20. In this scenario, three different visual acuities for a single eye would be recorded in one clinical note.

The vision assessed in an examination with the patient not wearing any glasses or contact lens correction, is recorded as “uncorrected visual acuity.” If the patient is wearing glasses or contacts, it is recorded as “corrected visual acuity.” In a person with normal eyesight who does not need glasses, their vision without glasses (“uncorrected” visual acuity) is expected to be 20/20. In myopic (near-sighted) or hyperopic (far-sighted) patients who wear appropriate glasses and otherwise have a normal visual system, their vision with glasses (“corrected” visual acuity) would also be expected to be 20/20. If a person has an eye problem such as a cataract or diabetic eye disease, their “best corrected” vision glasses may be worse than 20/20.

Patients often present to an ophthalmologist’s office because of blurred vision, which may be due to the use of a lens prescription that is outdated for their eyes. It may also be due to an underlying disease of the eye that is limiting vision. In either situation, a test called refraction may be performed. Refraction (measuring for glasses) will measure the appropriate lens strength to focus light on the retina and determine the eye’s visual potential or best corrected visual acuity (BCVA). Clinically, it is the single BCVA for each eye that represents the maximal visual potential, and this value is of most interest to clinicians and researchers [1].

Patients with an eye disease such as cataract may see 20/100 with their old glasses. They may be subsequently refracted but may only be able to see 20/50 with the new lenses because the cataract partially blocks the vision. Technically, the BCVA can only be determined if a patient is refracted during the visit. In the preceding example, the BCVA is the same as the best documented visual acuity (BDVA), that is, 20/50. If the patient above was not refracted during that visit, the BDVA for that encounter would have been 20/100 and the BCVA would be unknown.

Sometimes a quick test such as the pinhole test can approximate the best refraction or BCVA, but is not as accurate as the “gold standard” of refraction. Also, in some office visits, no refraction or pinhole test is performed, so the only visual acuity is the “current” visual acuity, and the BDVA may or may not be equal or even close to the true BCVA. Therefore, while BCVA is the commonly used clinical term, when abstracting visual acuities from an EHR, BDVA is the appropriate terminology used.

In the example illustrated in Table 1 , a patient had three office visits to three different eye care providers over a span of 1 month. In the first visit it was noticed that the patient had blurred vision in both eyes and the patient was refracted. It was discovered that the patient’s right eye had a limited vision due to diabetic retinopathy and the left eye needed updated glasses. During this visit, the BCVA was found to be the same as the BDVA. During the second visit, the retina specialist did not refract the patient, but used a pinhole to estimate the BCVA. In this visit, the BDVA was close to, but slightly different than, the true BCVA, which was not determined as the patient was not refracted. During the third visit to an eyelid specialist, the specialist only checked the vision with the then used glasses and did not refract or pinhole as it was not relevant to the reason for this visit. In this case, the BDVA was “worse” in each eye, but that was due to the lack of attempt to measure or estimate the BCVA.

**Table 1:** Sample clinical encounters and corresponding BDVAs.

Visit					
A. First visit with doctor for new glasses					
	Vision with correction		Right=20/100		Left=20/40
	Manifest refraction		Right=20/60		Left=20/20
	*BDVA*		*Right=20/60*		*Left=20/20*
B. Second visit with specialist to evaluate retina problem					
	Vision with correction		Right=20/100		Left=20/40
	Pinhole		Right=20/70		Left=20/25
	*BDVA*		*Right=20/70*		*Left=20/25*
C. Third visit with eyelid specialist for eyelid lesion					
	Vision with correction		Right=20/100		Left=20/40
	*BDVA*		*Right=20/100*		*Left=20/40*

^a^ *BDVA:* best documented visual acuity.

A proper algorithm will assess all visual acuities in defined fields for an encounter and return the one with the best vision in each eye.

In the clinical setting in the United States, visual acuity is most commonly measured using a Snellen chart, where the patients view a standard set of letters at a distance equivalent to 20 ft. to determine their own visual acuity compared with what a “normal-sighted” individual would see at 20 ft. (ie, 20/20.) The numerator is the distance at which the test is performed and the denominator is the distance at which the smallest letter identified by the patient subtends an angle of 5 arc min [1]. A higher number in the denominator is indicative of worse vision, that is, 20/100 is worse than 20/20. Visual acuity is generally checked in each eye individually for diagnostic purposes. There are other standards used to determine visual acuity, such as metric Snellen equivalents or logarithm of the minimum angle of resolution (LogMAR). Jäger values (J1, J2, and so on) or font print size (8, 10, 12, and so forth) are used to test near visual acuity.

Recent work supports the use of data in EHRs for accurate and efficient identification of specific disease phenotypes [2-9]. The Electronic Medical Records and Genomics (eMERGE) consortium has demonstrated numerous successes identifying disease phenotypes. Past work specific to ophthalmology utilized a combination of approaches to identify cataract cases from EHR-based phenotyping of clinical notes [10]. However, despite the importance of visual acuity as a primary measurement of how well a patient can see, no standard method exists for the rapid and accurate extraction of BDVA from EHR notes.

This paper describes an algorithm we developed to capture distance visual acuity data from ophthalmology EHR clinical notes. We applied the algorithm to 295,218 patient records in Northwestern Medicine’s Enterprise Data Warehouse (NMEDW). We then compared our detection method to a chart review of a random sample of 100 patient notes under the direction of a board-certified ophthalmologist to test accuracy.

## Methods

### Algorithm Development

Within the Northwestern Ophthalmology clinics, the EPIC EHR (EPIC Systems Corporation, Madison, WI) has been in use since 2007. The structured visual acuity (“Snellen–Linear”) field in the EPIC EHR allows for discrete abstraction of the results that are entered by the provider. There are three different standard units that can be used while designating the results for the visual acuity examination (Snellen, Jäger, and font print size). With the current version of EHR, visual acuity is entered as a free text option that allows the provider to choose to manually type in the results or choose from a drop-down menu. As a result, a large variety of responses can be entered in various visual acuity sections. In total, we identified 5668 unique responses, all of which we mapped back to a standard Snellen visual acuity notation from the list in Textbox 1.

**List of visual acuities used in algorithm development**

- 20⁄10
- 20⁄20
- 20⁄25
- 20⁄30
- 20⁄40
- 20⁄50
- 20⁄60
- 20⁄70
- 20⁄80
- 20⁄100
- 20⁄125
- 20⁄200
- 20⁄400
- CF (counting fingers)
- HM (hand motion)
- LP (light perception)
- NLP (no light perception)
- LP (light perception)

Visual acuity measurements can be recorded in at least eight structured fields within our EHR note for each eye. In our EHR, a separate visual acuity can be measured for each eye with or without correction, with a pinhole device, refraction before dilation drops, refraction after dilation drops, autorefraction, and near vision with or without correction.

To further complicate the data, while visual acuity is recorded in defined fields, it is entered as free text, making a direct abstraction less meaningful as a single measurement could be recorded in a variety of different ways. For instance, providers could often write other clinical information in the visual acuity field that may be helpful in future clinic visits. Examples of responses entered included: “20/20 slow,” “after waiting 1 min 20/20 in lighted room,” “20/60 w/head tilted down,” and “20/60 blinking with ointment.”

We extracted these data from our NMEDW using Structure Query Language (SQL). This language allows for the manipulation of the data in a convenient fashion and is the standard for most clinical databases. SQL allows for “keyword” searches where one can designate that a result must include a certain text string. All of the responses that included these were then manually mapped to one of the visual acuity categorizations in Textbox 1.

To address the fact that the 5668 unique responses found in the EHR do not represent every possible future input value, we developed a mechanism to categorize text not currently in the vocabulary list. It employed string searches for known visual acuities that were initially entered in the “visual acuity” structured field from the EHR notes. This was accomplished by taking all visual acuities listed in Textbox 1. The algorithm only used this method if it came across a result that could not be mapped back to a previously categorized response, as the human curated vocabulary was considered the “Gold Standard.”

**Figure 1:**
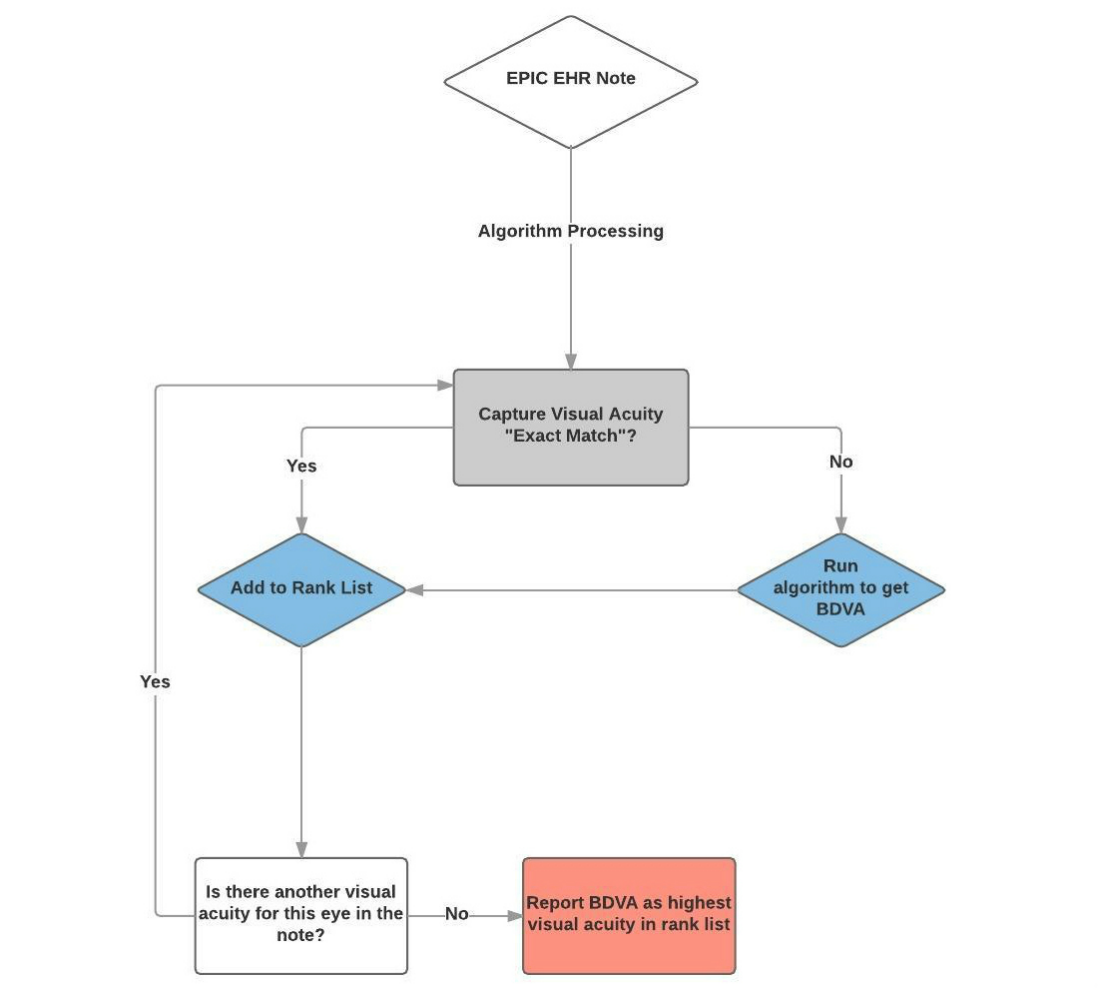
Algorithmic Determination of Best Documented Visual Acuity.

**Figure 2:**
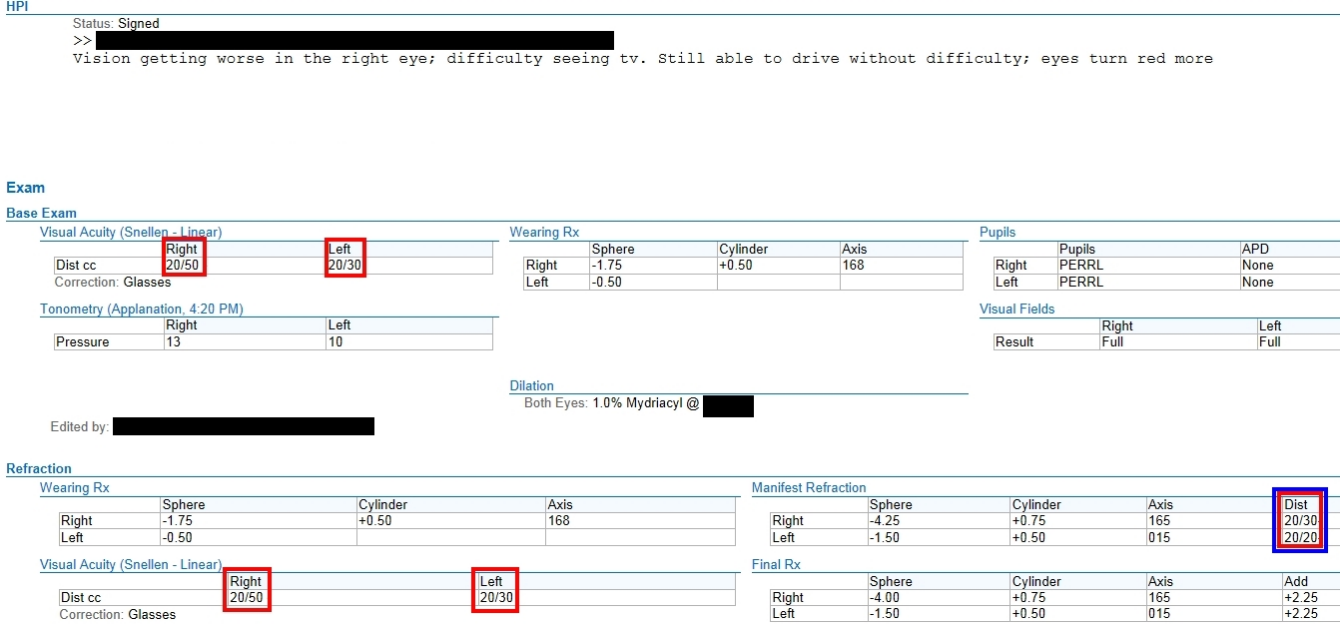
Screenshot of EPIC EHR provider input. Red Box outlines all fields containing visual acuity data (Right Eye: 20/50 and 20/30. Left Eye: 20/30, 20/20. Blue Box outlines what the algorithm detected as BDVA for each eye (Right Eye: 20/30, Left Eye: 20/20). ©2016 Epic Systems Corporation. Used with permission.

**Figure 3:**
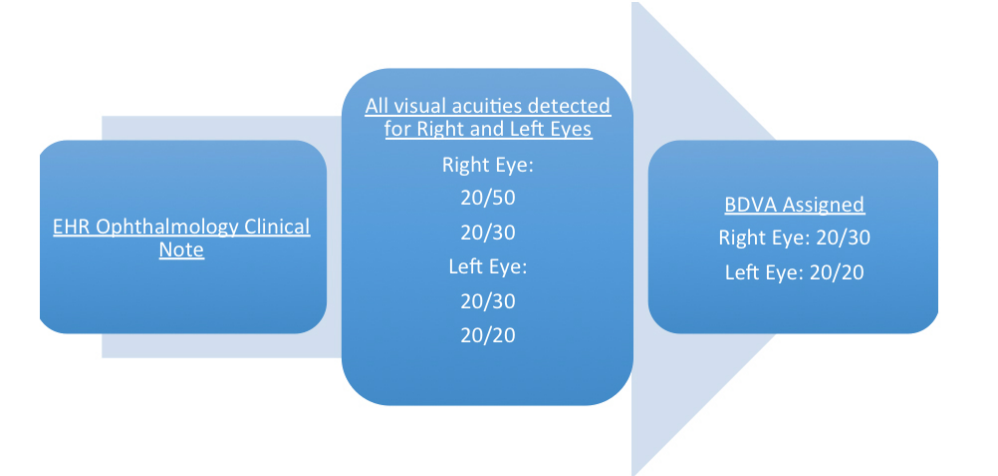
Flow diagram for algorithmic processing of the ophthalmology EHR note in Figure 2.

Visual acuities were then ranked in terms of best to worst as designated by their numeric representation. For example, the categorized result of 20/10 was ranked number one, 20/20 was ranked number two, and so on. This ranking allowed for additional coding to determine which visual acuity was the best for a particular patient note (Figures 2 and 3). All codes used for this study are currently under publication and will be later available at https://phekb.org for open use. Figure 1 illustrates the algorithm’s acuity mapping and ranking logic. Figures 2 and 3 detail an example of a BDVA determination from a clinical note.

### Data

We extracted the data from the NMEDW. The NMEDW is a joint initiative across the Northwestern University Feinberg School of Medicine and Northwestern Medicine. Its mission is to create a single, comprehensive, and integrated repository of all clinical and research data sources on the campus to facilitate research, clinical quality initiatives, healthcare operations, and medical education. The study began in early 2007 as this was the year when the ophthalmology clinic transitioned fully to an EHR.

The data for this study was obtained from the Northwestern Medicine Department of Ophthalmology adult outpatient ambulatory clinic visits at Northwestern Memorial Hospital, which uses the EPIC EHR. All patients aged between 18 and 89 years were included in the study. Additionally, all notes where a record included any measurement of a visual acuity (Snellen–Linear) were used to develop the algorithm. There were a total of 298,096 clinical notes from the Ophthalmology clinic between January 1, 2007, and December 31, 2014. Of these, 295,218 notes from 57,317 unique patients had at least one visual acuity measurement recorded in the chart and were therefore included in the analysis.

In order to evaluate the accuracy of the results of the algorithm, two reviewers, an ophthalmology attending physician and a medical student (PB, MM), independently reviewed 100 additional ophthalmology clinical notes and documented BDVA for each eye. For internal validation, a proper correlation was found between the two reviewers every time.

These BDVAs were then compared with those generated by the algorithm. Using clinician chart review as a gold standard, we evaluated the accuracy for our algorithm.

The protocol was approved by the Northwestern University Institutional Review Board Office in Chicago, Illinois.

## Results

**Figure 4:**
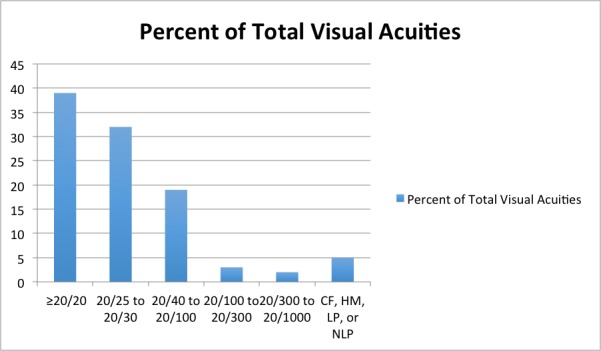
Graph depicting frequency of visual acuity detected within EHR notes by ranges (CF=Count Fingers, HM=Hand Motion, LP=Light Perception, NLP=No Light Perception).

About 295,218 ophthalmology clinical notes were found to have visual acuity data present. This represented 57,317 unique patients who had at least one eye examination for which visual acuity was captured. The overall average age of patients in this study was 57.6 years (range of 18–89 years). Most visual acuities detected in patients were 20/100 or better (86.2%; Figure 4); “20/20” was the most common visual acuity recorded (38.7%), followed by “20/25” (18.9%).

For each clinical note, there was an average of 1.48 and 1.49 visual acuity recordings for every right and left eye respectively, with a range of 0–7 acuities for each eye. Of the 295,218 clinical notes, 54% (158,786) had more than one visual acuity recorded for either the right or left eye. There were 5668 unique responses recorded in any of the defined visual acuity fields.

**Figure 5:**
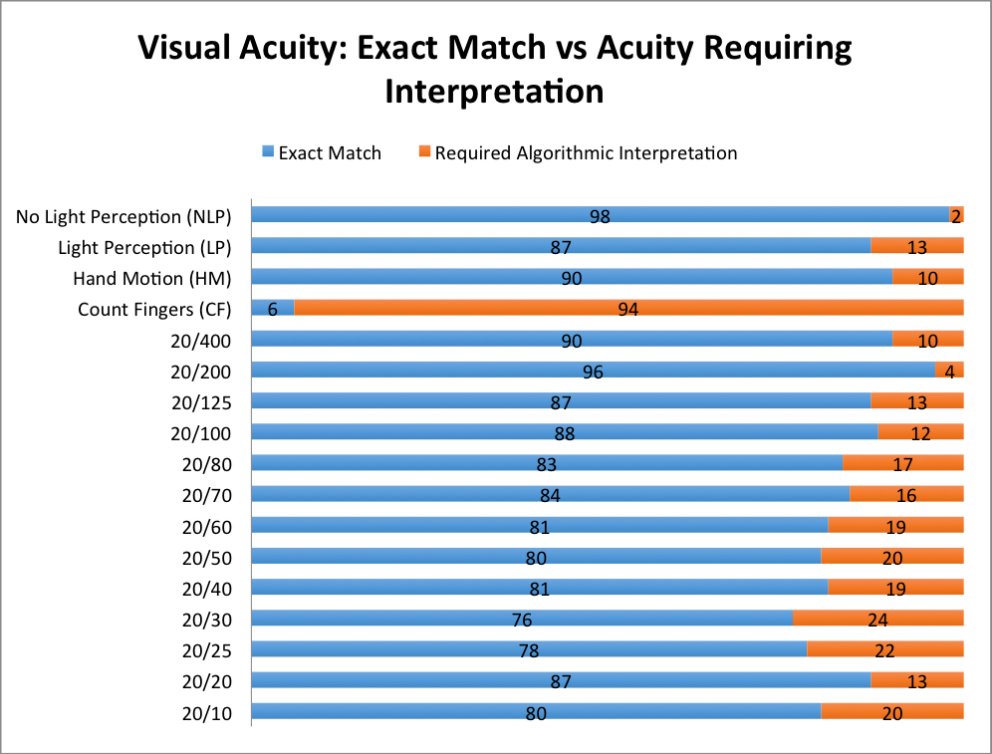
Visual Acuity as detected by algorithm.

When examining specific documented Snellen visual acuity values, approximately 80% of the time there was an exact match of the documented visual acuity when compared with the Snellen values in Textbox 1. The breakdown for each Snellen equivalent of exact match versus those acuities requiring interpretation by the algorithm is shown in Figure 5.

A random sampling of 100 patients (200 eyes) for which visual acuity was captured was used for a clinician chart review, and was conducted in a fashion similar to previously published work [10]. The BDVA noted by the clinicians was compared with the value captured by the algorithm. The algorithm was found to have an overall accuracy of 99% (99% right eye; 99% left eye), as shown in Table 2. Visual acuities documented in areas of the chart other than the structured visual acuity fields, such as the “History of Present Illness” portion of the clinical note, accounted for two (1.0%) instances of error.

**Table 2:** Chart review results of BDVA algorithm.

Total number of patients reviewed	100
Total number of eyes	200
Right eye accuracy	99%
Left eye accuracy	99%
Overall accuracy	99%

^a^ *BDVA:* best documented visual acuity.

## Discussion

We created a unique algorithm to accurately determine best documented distance Snellen visual acuity data from EHR systems using electronic ophthalmology clinical notes. This algorithm was used on a large-scale data repository of 295,218 notes and was validated comparing the results to a manual chart review of 100 clinical notes. The algorithm accurately detected visual acuity in 99% of cases.

### Principal Findings

Just as with visual acuity, there are numerous components of the medical record note (such as chief complaint, smoking status, allergies, and so forth) that may or may not contain completely “structured data,” and are not easily captured. The accurate representation of quantitative traits from EHR notes is often overlooked due to difficulty with how they are documented within the EHR (often in free text), or assumption that these data are implicit within a clinical diagnosis. Given these challenges, related methodology to our work has necessarily been developed for other measures, such as detection of cataract cases [10] and adult height [4] from EHR notes. Numerous studies attempt to capture these in accurate and efficient ways, with varying results [11-14]. Our work, to the best of our knowledge, represents the first attempt at analyzing and capturing best documented visual acuity from electronic ophthalmology notes. This effort will allow us to perform patient centered outcomes research from the electronic health record. Our future work will center on comparative effectiveness research with BDVA changes for various treatments of macular degeneration, diabetic retinopathy, and cataract surgery just to name a few. Additional work to define EHR-based phenotyping of quantitative traits like BDVA can enable higher throughput association studies [15-20].

### Limitations

There are limitations to our algorithm. First, with this method, it is only possible to categorize responses retrospectively and maintain complete confidence that they will be properly categorized. Any algorithm that searches free text may have difficulty deciphering it (eg, transposing the letter “O” for a “zero”). As visual acuity is captured as free text, a physician could enter a result that has never been used before and would not be captured by the current grouping method. We added more flexible rules, such as our alternative detection method, which could be put in place to attempt to categorize results prospectively but there is a potential for it to be inaccurate. Instead, it is likely that this method will require ongoing maintenance to maintain complete confidence.

Second, this algorithm was developed and tested using visual acuity values found in NMEDW and based on one EHR system. The algorithm currently searches in the “visual acuity” section of the EPIC EHR note. Should visual acuity be documented elsewhere, such as a descriptive phrase in the history or assessment, it will not return a result; however, in our study this occurred in less than one percent of visual acuity notes audited. While this is a potential limitation, other EHR systems are known to store data in a similar defined fields fashion, increasing the potential generalizability of our algorithm at other institutions and EHRs [21,22]. The application and use of our algorithm at different clinical sites, as well as on different EHR platforms, will be the focus of future work.

While this is a representative sample of the Snellen distance visual acuity measurements, it may be necessary to adjust the algorithm for other types of visual acuity measurement systems (such as logMAR, ETDRS, metric scales, and so on), or when serving different patient populations such as pediatric populations or low vision patients. Our algorithm is flexible and can be easily modified by incorporating results from site-specific chart reviews. All codes used for this study are currently available upon request to the corresponding author. As visual acuity is a primary marker of assessing visual health, this research represents a pivotal first step in making ophthalmology electronic medical notes easily accessible for research purposes.
